# A Study on China’s Tobacco Taxation and Its Influencing Factor on Economic Growth

**DOI:** 10.3389/fpsyg.2022.832040

**Published:** 2022-02-24

**Authors:** Shuang Zhao

**Affiliations:** School of Economics, Inner Mongolia University for Nationalities, Tongliao, China

**Keywords:** economic growth, population aging factor, logarithmic average weight decomposition method, sustainability, health psychology

## Abstract

Tobacco is a significant product providing considerable economic benefits to countries worldwide, while its increased consumption causes health and socio-economic losses for smokers and non-smokers. This paper constructs a decomposition system of tobacco taxation: the population aging factor is included in the influencing factors of personal tax, and personal tax revenue is regarded as the product of tax structure, macro tax burden, regional economy, reciprocal aging, and the elderly population. This article conducts an empirical study on the relationship between taxation and economic growth. The estimated coefficients of business tax and corporate income tax are significant at the significance level of 0.1, with a consumption tax and time-variable coefficients reporting a 0.02 level of significance. The T statistic value and the explanatory degree of the variables involved in the model to the explained variables are also very high, reaching more than 95%. We find that increasing the macro tax burden negatively impacts economic growth. Therefore, the study suggests that for fostering the industry’s economic growth, the country needs to ensure the optimal macro tax burden of 17.5%, with different types of taxes influencing economic growth. Personal tax reform should pay attention to the phenomenon of aging, adjust the tax structure to increase personal tax income, provide policy support and guarantee for the elderly labor force, and encourage the re-employment of silver-haired people to alleviate the adverse impact of aging on taxation.

## Introduction

Tobacco is a particular product. Compared with ordinary commodities, because its ingredients contain nicotine, smokers are easily addicted and become dependent on tobacco, resulting in lower elasticity of demand for tobacco products. In addition, its consumption behavior also has incomplete information, which will cause negative externalities and pathogenicity in the consumption process. Compared with drugs, tobacco is addictive and causes less health damage, and the symptoms after addiction are controllable. Therefore, all countries have adopted an attitude of “restricting tobacco use.” This has led to such a result: on the one hand, tobacco production can bring huge economic benefits to society and the government; on the other hand, tobacco consumption will lead to immeasurable socio-economic loss.

In today’s modern era, several global industries have competed in the international market (Abdullah et al., 2021), with the tobacco industry gaining economic dominance. The tobacco industry occupies an important position in China’s economic development (Yeh et al., 2017). China’s tobacco product production, consumption, and several smokers have long ranked first globally, and the total output value has continued to maintain a momentum of rapid growth (Verguet et al., 2021). In the past 10 years, the annual growth rate of cigarette output value has been about 10%. It can be said that the tobacco industry in China has brought huge economic benefits. In the annual fiscal revenue of the Chinese government, tax revenue from the tobacco industry has always maintained a relatively high share, contributing nearly one-tenth of the country’s fiscal revenue (Jain et al., 2020). Taking 2019 as an example, the tobacco industry contributed 915.61 billion yuan in taxes and fees to the country’s income. At the same time, tobacco companies account for about one-third of the top 100 taxpayers in China (Ho et al., 2018). Tobacco tax involves various types of taxes established for tobacco, such as tobacco leaf tax, tobacco excise tax, value-added tax, corporate income tax on tobacco production, etc. Still, tobacco excise tax accounts for 50 to 60% of the share, making it a government development.

In recent years, ensuring stakeholder interest has become essential for economic progression (Ajaz et al., 2020). Therefore, for the improvement of countries environment (i.e., CSR) (Shah et al., 2021) and people’s health, the control of tobacco consumption has become the consensus of all countries in the world (Nargis et al., 2021). To reduce the harm of tobacco consumption to the human body and reduce the consumption of various social resources caused by tobacco consumption, most countries have adopted government control. However, the theoretical research of scholars and the experience of tobacco control by government departments around the world show that the use of tobacco excise tax for regulation is the most effective way recognized internationally (Du et al., 2019; Yu et al., 2020). On the one hand, the tobacco consumption tax is an indirect tax. It has the general attributes of indirect tax and can form a price transmission mechanism. Through taxation, the retail price of tobacco products can be increased, and curbing tobacco consumption can be achieved. Part of the income brought by the levy of tobacco excise tax can be fed back to the tobacco control campaign, which in turn forms a benign tobacco control mechanism (Kostova et al., 2017). Taxation structure and taxation level are the two most important parts of taxation system reform. Through analyzing the transmission mechanism of taxation level and taxation structure to economic growth, combined with empirical research, we can summarize the direction and measures of taxation system reform and provide for optimization of taxation system.

Under the background that tobacco consumption has become a huge threat to global health, raising tobacco excise tax has been proven by international practice as the most effective and cost-effective tobacco control method. This is because, compared with other tobacco control measures, taxation has a wider scope and raises huge financial revenue for the government while effectively controlling tobacco.

With the in-depth research of domestic and foreign experts and scholars on tobacco consumption, policy suggestions for the reform of tobacco tax are also constantly put forward. Through induction and sorting, it is found that scholars mostly focus on empirical analysis, studying the elasticity of consumer demand for tobacco, the impact of tobacco tax increases on tobacco consumption, and estimating the direct and indirect costs caused by smoking—however, few studies on the overall system design of tobacco excise tax.

Therefore, based on previous scholars’ research, this paper intends to study the specific design of each tax system element of tobacco consumption tax from the perspective of tax system optimization through the combination of qualitative and quantitative research methods, inductive analysis, and comparative analysis. From the perspective of theoretical construction, this paper has certain theoretical significance for making up for the relevant deficiencies and improving the entire taxation theoretical system.

The mechanism of the optimization of the tobacco consumption tax system is mainly carried out from two perspectives: how to achieve the tobacco control target of the tobacco consumption tax and how the tax system elements are designed to achieve the tobacco control target. The analysis of the former’s taxation goal provides an optimization direction for the design of tax system elements. In contrast, the latter’s discussion guarantees the realization of the former’s taxation goal. At the national level, we decompose the personal income tax income of the total tobacco company employees. The results of the LMDI decomposition show that the aging effect has an eroding effect on the individual tax income, which seriously hinders the growth and development of the individual tax income and often causes the individual tax income. The negative growth of the company partially offsets the driving effect of other effects on personal tax revenue. Although there are differences in the contribution rate of the various decomposition effects between regions to the total effect, economic effects are always the main reason for the growth of individual taxes in each region.

Tax structure and the macro tax burden also determine how individual tax revenues grow. There is a significant gap between China’s tax structure, macro tax burden, and international levels. Policymakers should also focus on these two factors when making decisions on individual tax reform. The number of elderly practitioners continues to increase, and the expansion of the elderly population has also contributed to the increase in personal tax revenue. Perhaps policy support in elderly employment can also alleviate the tax pressure caused by aging. We analyze and study the impact of Chinese taxation on economic growth by empirically testing the impact of different taxes or tax types on economic growth. Value-added tax, consumption tax, and personal income tax for tobacco company employees have a negative impact on China’s per capita GDP; business tax and corporate income tax have a positive impact on China’s per capita GDP.

The rest of this paper is organized as follows. Section “Study Background” discusses the research background. Section “Mechanism Analysis of Optimization of Tobacco Consumption Tax System” analyzes the mechanism of tobacco excise tax optimization. Section “Lmdi Decomposition of Personal Income Tax for Tobacco Company Employees With the Introduction of Population Aging Factors” analyzes the personal income tax of tobacco company employees by introducing population aging factors. Section “Empirical Test of the Impact of Tobacco Tax Structure on Economic Growth” empirically examines the impact of tobacco tax structure on economic growth, with Section “Conclusion” concluding the research findings by proposing significant implications for future researchers.

## Study Background

Regarding the impact of taxation levels on economic growth, foreign scholars have conducted a lot of research through theoretical derivation and empirical analysis (Postolovska et al., 2018). The Keynesian school advocated that the state intervene in the economy and ensure the government’s efficient operation by increasing taxation levels (Chen et al., 2019). The monetary school points out that tax rates should be used discretionarily during the economic cycle to resolve economic problems (Wang M. et al., 2019). The government does not recommend tax cuts as it believes that tax cuts will increase the number of funds in circulation, increase social purchasing power, and further worsen inflation. However, the supply school believes that two different tax rates can achieve the same tax effect. It advocates a lower tax rate, ensuring fiscal revenue and promoting economic growth. In terms of scale changes, related scholars believe that China’s macro taxation level is steadily rising, and within the optimal range, the current taxation level has maximized economic growth (Zhi et al., 2019). Researchers designed the measurement relationship between tax burden and economic growth under the premise of continuous economic optimization and calculated that China’s optimal tax burden is 16.7%, and the current tax level has exceeded this ratio (Álvarez et al., 2020). Some scholars also believe that this result is due to the inconsistent caliber of the tax level calculation (Kipkorir et al., 2019). Therefore, the researchers divided the tax burden and analyzed that China’s small-caliber macro taxation level is in harmony with China’s economic development. Still, based on the medium and large-caliber calculations, the tax burden has hindered the healthy development of the economy.

Domestic research on the impact of taxation levels on economic growth models has various forms, which is worth learning. Still, different conclusions have reduced the reference value of these academic papers. The reason may be the choice of models, data processing, or professional restrictions. First of all, the choice of data, most of the literature adopts local taxation data when studying the provincial taxation panel. However, since the taxation system reform, the proportion of local taxes is about 40%, which does not reflect the true taxation level. Secondly, there is an inherent correlation between taxation and economic growth and should be considered comprehensively. Finally, the reform of the tax system will affect the changes in the proportion of various taxes in the total tax level, which in turn affects the level of taxation and economic growth, and changes in the tax system structure will also have an impact on the level of taxation (Amarasinghe et al., 2018; Ren et al., 2020; Zheng et al., 2021).

Relevant scholars have calculated through welfare effects and have given that the welfare harm caused by the business tax is far greater than the harm caused by value-added tax, but also pointed out that small and micro enterprises should be exempted from value-added tax. They also advocated the “deduction of value-added tax in the service industry” (James et al., 2019). In addition, related scholars have studied the impact of value-added tax and corporate income tax on the company’s investment value and found that the investment subsidy effect of value-added tax is greater than the negative effect of corporate income tax. Once the income tax is reduced, the positive effect of the value-added tax will be greater (Wang R. et al., 2019). Regarding the consumption tax, the researchers pointed out that although the country has repeatedly revised the consumption tax base and tax rate, the adjustment effect of the consumption tax has never been obvious. Relevant scholars have also pointed out the need to further optimize consumption tax from the output in their research (Mori and Tsuge, 2017). The tax object can be appropriately expanded, and luxury, high-consumption, low-yield, and environmentally polluting commodities are included in the scope of taxation. Regarding direct taxes, relevant scholars have analyzed the effects of corporate income tax on economic growth and income distribution and concluded that corporate income tax would increase social capital and labor and expand the overall scale of the economy.

From the perspective of tax burden shifting and ultimate fate, the research results of related scholars pointed out that tobacco consumption accounts for a relatively high proportion of the total consumption of low-income people, which is common in all countries in the world. If the tobacco consumption tax is increased, the burden of the tobacco consumption tax will be increased. Retirement will also increase, which is a departure from the principle of tax fairness. Researchers believe that although tobacco consumption is harmful to physical and mental health, tobacco products are addictive, and their price elasticity is small. The increase in tax burden can be effectively transferred to consumers, thus playing a role in restraining tobacco consumption (Guo and Quan, 2020).

## Mechanism Analysis of Optimization of Tobacco Consumption Tax System

### Realization of the Taxation Goal of Tobacco Excise Tax

China’s cigarette price system includes four major parts: ex-factory price, transfer price (tax-calculated price), wholesale price, and retail price. The transfer price is the tax base of the China Tobacco Consumption Tax. It refers to the cigarette transaction price signed between the cigarette manufacturer and the purchaser in the production process. This price is the basis of the ex-factory price, determined by the State Tobacco Monopoly Administration and the tax Jointly decided by the agencies. The wholesale price is the price formed after adding a specific adjustment gross profit rate (the profit rate between the cigarette adjustment price and the wholesale price) based on the allocation price. Finally, the retail price of cigarettes is based on the wholesale price plus the prescribed gross margin (the profit margin between the wholesale price and the retail price of cigarettes). The State Tobacco Monopoly Administration regulates the adjusted gross margin and the gross margin.

Because the high-income group and the group with high tobacco addiction have less elastic demand for tobacco, the affordability of tobacco is higher, or the consumption habits are relatively stable, the product substitution caused by the increase of tobacco consumption tax is not high, and the consumption volume has remained stable. Even for high-income earners, due to the basic national conditions of China’s social “respect for tobacco” and “send tobacco,” the increase in tobacco prices may also promote their purchase of high-end tobacco. On the other hand, for low-income earners and groups with small addictions, although tobacco consumption has decreased, the unit price increase brought about by tax increases can make up for the fiscal revenue loss caused by the decrease in tobacco sales.

**FIGURE 1:**
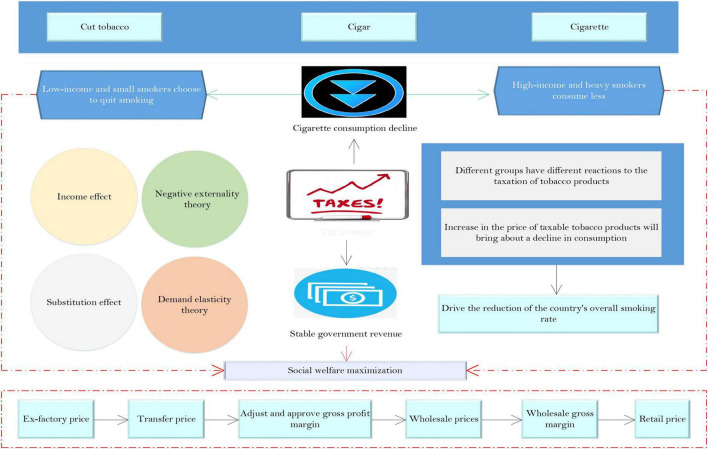
Conceptual framework diagram of the impact of raising the tobacco excise tax on tobacco consumption and government revenue.

As shown in Figure 1, based on ensuring that the increased tax revenue passed to the final retail price, that is, based on the tax price linkage, the method of raising taxes on tobacco products reasonably is not only in line to control tobacco consumption, but also meeting the goal of stabilizing fiscal revenue. Therefore, this factor ultimately maximizes social welfare.

### Mechanism Analysis of Optimization of Tobacco Excise Tax System Elements

Over the years, China has potentially focused on increasing its production level (Mohsin et al., 2021) rather than the consumption pattern. Consumption tax is an important economic means of national macro-control. The determination of its taxation scope reflects the tax policy and guiding ideology of a country or region in a specific period and directly affects the changes in related consumer behavior. For tobacco consumption tax, the scope of its collection should also be updated and adjusted in a special period. China’s current tobacco consumption tax collection design only imposes consumption tax on three types of traditional tobacco products: cigarettes, cigars, and cut tobacco. The alternative products of cigarettes, namely new tobacco products, have not yet been included in the tax collection and management system. Under the current background of levying taxes on traditional cigarettes, if new tobacco products such as e-cigarettes are not taxed, smokers will eventually switch to the field of e-cigarettes based on the income effect and substitution effect, which will not only affect the high value of traditional tobacco products. Taxation has an impact and runs counter to the heavy taxation goal of tobacco control.

**FIGURE 2:**
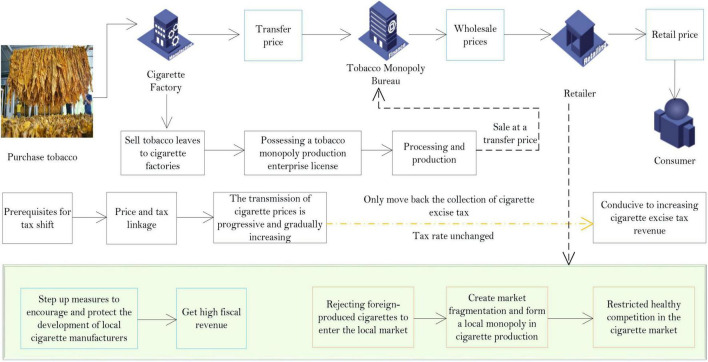
The process of the tobacco industry.

The collection of cigarette excise tax includes three links, namely, production, wholesale, and retail. Since China implements a tobacco monopoly system, this article only demonstrates the advantages and disadvantages of the three links in this context. Before comparing the various collection links, we must understand the specific operation of the tobacco industry. The process of the tobacco industry is shown in Figure 2.

As far as the production link is concerned, the current tobacco consumption tax in most countries globally is levied in this link, and China initially implemented a single levy on the production link. This is because most tobacco production companies are general taxpayers, and tax authorities can control them through a series of methods such as input tax, output tax, and equipment capacity. Hence, it is easy to obtain relevant tax data. Tobacco companies have strong control and can obtain higher tax revenues at extremely low taxation costs; taxation at the source is more efficient.

However, behind the theory of efficient collection of production links, there is a problem of local protectionism in collecting tobacco taxes in practice. Although the consumption tax belongs to the central government’s fiscal revenue and will eventually be paid to the state, because the consumption tax is levied at the place of production, the urban maintenance and construction tax and education surcharge calculated with the consumption tax as the tax base belongs to the local tax revenue. In addition, according to the tax refund calculation formula determined by the tax-sharing system, the huge tobacco consumption tax levied at the production area constitutes one of the bases of the central tax refund. Therefore, before these two “preferential policies,” on the one hand, local governments that mainly produce cigarettes will increase measures to encourage and protect the development of local cigarette manufacturers from obtaining high fiscal revenue. When producing cigarettes, local governments will choose to deny them access to the local market for fiscal revenue, which will result in market separation and form a local monopoly in cigarette production.

**FIGURE 3:**
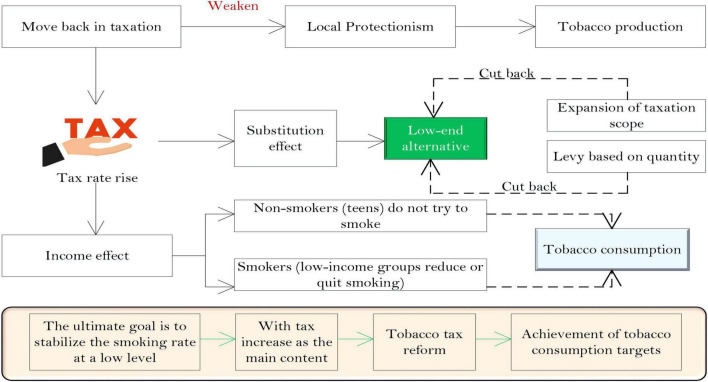
Optimization framework of tobacco consumption tax system.

In summary, levying a cigarette excise tax only in the production process runs counter to the country’s total tobacco production capacity restriction goal and restricts healthy competition in the cigarette market. This situation is particularly prominent in provinces that rely heavily on tobacco tax revenue. The optimization framework diagram of the tobacco consumption tax system is shown in Figure 3.

## LMDI Decomposition of Personal Income Tax for Tobacco Company Employees With the Introduction of Population Aging Factors

### Decomposition of LMDI of Tobacco Company Employees’ Personal Income Tax Under the Factor of Population Aging

To reasonably divide and decompose the personal income tax of tobacco company employees, assume that the personal income tax of the total tobacco company employees in the region comes from the four regions of East, Central, West, and Northeast. Let PIT be the personal income tax of tobacco company employees; PITi is the individual tobacco company employees in the i region. T is the total tax revenue of the region, Ti is the total tax revenue of the i region; Y is the total value-added, Yi is the total value added of the i region; TP is the total population of the region, TP_*i*_ is the total population of the region; OP is the size of the elderly, OP_*i*_ is the size of the elderly population in area i, the personal income tax income of Chinese tobacco company employees can be expressed as:


(1) $$PIT=\prod_{i}\left(\frac{TP_{i}}{OP_{i}}•\frac{Y_{i}}{1-TP_{i}}•\frac{T_{i}}{1-Y_{i}}\right)$$

Among them, S_*i*_ is the tax structure of region i, M_*i*_ is the macro tax burden of region i, Y_*i*_ is the regional economy of region i, and A_*i*_ is the regional aging factor: the ratio of the total population to the elderly population can be regarded as the reciprocal of aging. O_*i*_ is the size of the elderly population in area i. Therefore, the total personal income tax of employees of tobacco companies in the country can be regarded as a chain index of tax structure, macro tax burden, regional economy, aging factors, and the size of the aging population. To measure the effectiveness of the above factors on the changes in the total personal income tax of the employees of the tobacco companies in the region, this paper uses the LMDI decomposition method to study the changes in the individual tax in each region. It examines the regional individual tax income changes from the two aspects of the development speed and growth of the individual tax. The incremental decomposition of personal income tax income of tobacco company employees can be expressed as:


(2) $$\Delta PIT=\prod_{i}\left(O_{i}^{t}•S_{i}^{t}•A_{i}^{t}•M_{i}^{t}•Y_{i}^{t}\right)$$

The decomposition effects are, respectively, expressed as:


(3) $$\Delta PIT_{S}=\prod_{i}L\left(\begin{array}{cc} PIT_{i,t} & PIT_{i,0} \end{array}\right)•\ln\left(s_{i,t}/s_{i,0}\right)$$

(4) $$\Delta PIT_{M}=\prod_{i}L\left(\begin{array}{cc} PIT_{i,t} & PIT_{i,0} \end{array}\right)•\ln\left(M_{i,t}/M_{i,0}\right)$$

(5) $$\Delta PIT_{Y}=\prod_{i}L\left(\begin{array}{cc} PIT_{i,t} & PIT_{i,0} \end{array}\right)•\ln\left(Y_{i,t}/Y_{i,0}\right)$$

(6) $$\Delta PIT_{A}=\prod_{i}L\left(\begin{array}{cc} PIT_{i,t} & PIT_{i,0} \end{array}\right)•\ln\left(A_{i,t}/A_{i,0}\right)$$

(7) $$\Delta PIT_{O}=\prod_{i}L\left(\begin{array}{cc} PIT_{i,t} & PIT_{i,0} \end{array}\right)•\ln\left(O_{i,t}/O_{i,0}\right)$$

The additive decomposition component of each factor means that other factors remain unchanged, only the incremental change in the total personal tax income caused by the change of the factor. The multiplicative decomposition formula of individual income tax income of tobacco company employees is as follows:


(8) $$R_{PIT}=\frac{\prod_{i}\left(A_{i}^{t}•M_{i}^{t}•Y_{i}^{t}•O_{i}^{t}•S_{i}^{t}\right)}{\prod_{i}\left(A_{i}^{0}•M_{i}^{0}•Y_{i}^{0}•O_{i}^{0}•S_{i}^{0}\right)}$$

### LMDI Decomposition Results of Changes in Personal Income Tax Income of Tobacco Company Employees

Regional macroeconomic conditions and development levels are different. How will the personal income tax income of employees of tobacco companies in China change? When other factors are fixed, what impact does the national tax structure, macro tax burden, regional economy, aging, and the size of the elderly population have on personal tax revenue? This article will discuss this issue in detail. The individual income tax of employees of tobacco companies across the country can be regarded as a summary of individual tax income at the regional level.

Analyzing Table 1 shows that China’s total personal tax revenue has increased year by year. From 2008 to 2020, personal tax revenue has increased by 589.725.8 billion yuan. It has a driving effect, among which the economic effect is the most significant. The increase in personal tax revenue caused by the economic effect is 413.74 billion yuan, far exceeding the driving effect of the other three effects. China’s tax structure and macro tax burden increased only slightly. From 2008 to 2020, the proportion of individual tax revenue to total revenue increased by 0.31%. At the same time, the ratio of tax revenue to total output also rose slightly, increasing by 3.4%, respectively. It caused an increase of 21.26 billion yuan and 90.57 billion yuan in personal tax revenue.

**TABLE 1:** Decomposition results of national individual tax revenue increment.

Years	Tax increment	Tax structure	Macro tax burden	Economic effects	Population aging effect	Elderly population size
2008–2009	254	−21	58	201	2	14
2009–2010	271	7	18	225	−65	117
2010–2011	543	−136	331	294	−1.8	53
2011–2012	301	14	−27	261	−38	99
2012–2013	241	−91	−6	279	8	47
2013–2014	547	26	91	337	409	324
2014–2015	692	58	200	401	112	−69
2015–2016	−165	−802	224	367	−204	248
2016–2017	500	162	−70	366	−230	276
2017–2018	632	211	35	367	−109	154
2018–2019	−1,006	537	8	415	−501	531
2019–2020	989	921	−422	467	−301	355

The scale effect of the elderly population has also actively promoted the growth of individual tax income. From 2008 to 2020, China’s elderly population has increased by 37 million, and the population effect has led to an increase of 119.61 billion yuan in individual tax income. The reason may be due to the rich human resources of the elderly in China. According to the “Survey Report on the Living Conditions of the Elderly in Urban and Rural Areas in China,” the number of working elderlies in China has increased rapidly since 1990. In 1995, the number of working people over 60 in China was 60.648 million. In 2019, this indicator increased to 92.353 million. In the same year, the working population aged 65 and above also increased by 13.954 million, an increase of 114% during the period.

Between 2008 and 2020, the ratio of China’s total population to the elderly declined, from 11.62 to 9.15, a decrease of 2.46, and the aging degree increased by 2.26% compared to 2008, which significantly hindered the growth of individual tax income. This reduced personal tax revenue by 55.55 billion yuan. Changes in the reciprocal of aging will bring about changes in tax revenue in the same direction. For the sake of brevity, the following are collectively referred to as aging effects.

Analyzing the decomposition results of individual tax revenue in each year shows that changes in the tax structure, macro tax burden, regional economy, and the size of the elderly population will bring changes in the same direction of tax revenue, and changes in the aging effect will change in the opposite direction of tax revenue. Taking the data from 2008 to 2011 as an example, China’s tax structure has been reduced year by year, and the ratio of individual tax revenue to total tax revenue has decreased year by year. This change has caused a negative growth in individual tax revenue. The change in tax structure in 2011 has reduced individual tax revenue by 14.16 billion yuan. In the same year, the macro tax burden increased by 1.87% compared with the previous year, which led to an increase of 34.39 billion yuan in personal tax revenue. The economic effect also contributed to an increase of 29.97 billion yuan in personal tax revenue. In the same year, the degree of aging increased compared with the previous year. The increase from 9.32 to 9.49% caused a decrease of 161 million yuan in personal tax revenue. Data from other years can also support this conclusion. The LMDI decomposition of the development speed of national personal tax revenue is shown in Table 2.

**TABLE 2:** Decomposition results of the development speed of national personal tax revenue.

Years	Personal tax development speed	Tax structure	Macro tax burden	Economic effects	Population aging effect	Elderly population size
2008–2009	1.25	1.1	1.14	1.22	1.10	1.13
2009–2010	1.24	1.12	1.11	1.23	1.07	1.15
2010–2011	1.33	1.05	1.24	1.25	1.13	1.12
2011–2012	1.21	1.10	1.01	1.21	1.02	1.14
2012–2013	1.11	1.12	1.04	1.25	1.03	1.06
2013–2014	1.32	1.15	1.08	1.29	1.05	1.21
2014–2015	1.18	1.15	1.11	1.25	1.05	1.17
2015–2016	1.09	1.20	1.21	1.22	1.32	1.04
2016–2017	1.11	1.31	1.11	1.26	1.12	1.15
2017–2018	1.40	1.12	1.31	1.11	1.22	1.19
2018–2019	1.30	1.14	1.13	1.22	1.12	1.17
2019–2020	1.26	1.17	1.11	1.28	1.24	1.08

Analyzing Table 2 shows that the growth rate of individual tax revenue has maintained above 10%. In 2011, the individual tax revenue growth rate was 1.34, and the growth rate reached more than 20%. The development momentum is good. From 2008 to 2020, the national personal tax revenue development rate will be 4.51, slightly lower than the eastern region’s personal tax development indicator. The economic effect caused the growth rate of personal tax revenue to be 182.1%, and the stimulating effect of the size of the elderly population on the personal tax revenue was the second, which caused the growth rate of personal tax revenue to be 36%. The pulling effect of the tax is relatively small, driving the growth rate of personal tax income to 5.6 and 25.7%, respectively. The aging effect affects the development rate of personal tax to 0.89, which means that the deepening of aging has caused a negative growth rate of 13% in personal tax revenue, which seriously hinders the development level of personal tax.

The decomposition of each year also shows that China’s tax structure and macro tax burden fluctuate year by year, and there is no stable trend. The two major fiscal effects on the promotion or suppression of individual tax revenue cannot be generalized. Their role depends on these two influencing factors. China’s economic growth is in good shape, and the economic effects continue to promote the growth of personal tax revenue. In 2014, the growth rate of personal tax revenue reached 9.5%, and the growth rate of personal tax revenue caused by the decline in the size of the elderly population was −8.2%. The deepening of the aging degree inhibits the growth and development of personal tax income. The annual decomposition results show that the degree of aging will cause a negative growth rate of personal tax income.

## Empirical Test of the Impact of Tobacco Tax Structure on Economic Growth

### Model Setting and Related Data Selection

This article will use the relevant data of a particular year as the analysis sample to estimate the above model. Because observing the macro tax rate during this period, it can be found that the tax rate is basically above 30%, with an average of about 37%. The economic system has undergone tremendous changes, and the tax system has also undergone new changes. Therefore, the macro tax rate has been directly reduced to about 20%. With the implementation of the tax-sharing system, the tax system has gradually improved and entered a stable stage. Therefore, this paper will use the data since the tax-sharing system as a sample for statistical analysis, which can better reflect the impact of taxation on economic growth.

This article will select five major taxes, namely value-added tax, business tax, corporate income tax, personal income tax for tobacco company employees, and consumption tax as explanatory variables. The control variable is set as China’s total investment. Generally speaking, a country’s GDP growth is driven by the troika of consumption, investment, and import and export. According to China’s current situation, imports and export are relatively small, and the consumption capacity needs to be improved. Investment is mainly used to drive GDP growth. And investment can be divided into inventory investment and social fixed asset investment. Since the investment in fixed assets of the whole society is the most favorable means of stimulating China’s economic growth, we will use the fixed asset investment data to replace the investment here. The explained variable is the per capita GDP value. In addition to the introduced control variable of total investment, we will introduce time t because the data designed by our model are all-time series, and we must consider the impact of time on the results. To eliminate the price impact, all explanatory variables, explained variables, and control variables used here will be calculated using actual price data, the real GDP per capita after the price factor is proposed. Finally, we will do a natural logarithm of all variables to prevent the occurrence of heteroscedasticity. Therefore, we set the specific regression model equation as follows:


(9) $$\begin{array}{c} \ln rjgdp=t+b_{0}\ln grsdy+b_{1}\ln zzs+b_{2}\ln yys\\ \;+b_{3}\ln xfs+b_{4}\ln qysds+\ln i+\delta \end{array}$$

**FIGURE 4:**
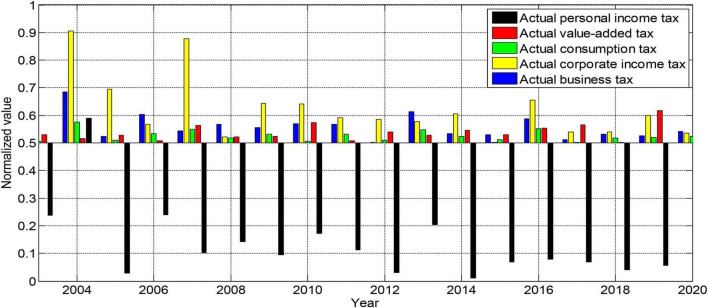
Normalized 2003–2020 data sample.

Where lnrjgdp is the logarithm of China’s per capita GDP, lnzzs, lnyys, lnxfs, lnqysds, and lngrsdy are the logarithms of China’s value-added tax, business tax, consumption tax, corporate income tax, and personal income tax for tobacco company employees. *t* is the time series, and δ is the white noise. On the one hand, China’s tax system structure has gradually stabilized after the tax-sharing system in 2003. On the other hand, to maintain consistency with the analysis data in the previous section, we will still select relevant data from 2003 to 2020 as the analysis object. The normalized 2003–2020 data sample is shown in Figure 4.

### Model Regression Analysis

Before performing the unit root test, it is necessary to determine the form of the variable. Usually, it is necessary to determine which form the variable belongs to by drawing the curve graph of the sequence. If the graph of a variable appears to fluctuate with a zero value, then the test type for this variable can be determined to have no intercept term and no time trend. A variable can be defined as having an intercept term if the variable’s graph appears to fluctuate from zero without a clear time trend. If the variable graph shows a clear trend in one direction over time, then the variable can be identified as a test type containing both an intercept term and a time trend term.

**FIGURE 5:**
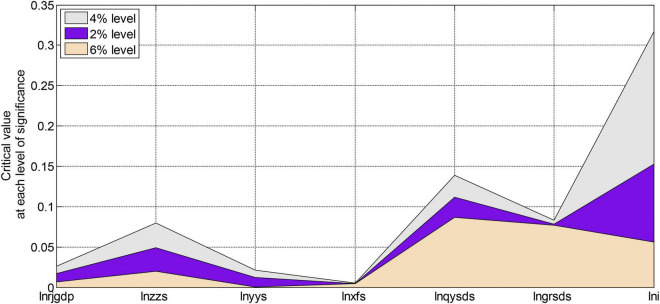
Critical values at each level of significance.

**FIGURE 6:**
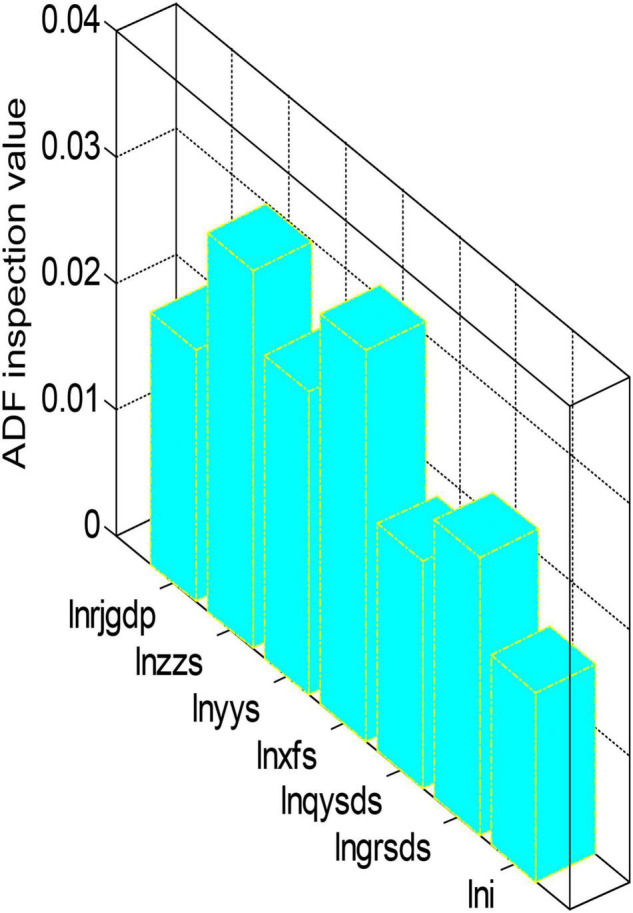
ADF unit root stationarity test results.

Since the sample data we used here is still a time series, we must first perform unit root verification on all variables in the model before estimating the model to prevent the occurrence of spurious regression. Here we still choose to use the ADF method to carry out unit root tests. Using EVIEWS6.0 software, the ADF unit root stationarity verification results of all variables of the model are shown in Figures 5, 6.

**FIGURE 7:**
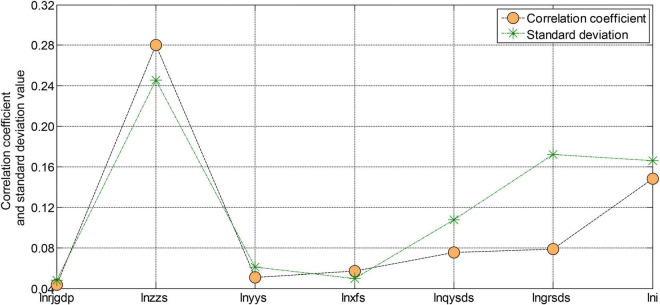
Correlation coefficient and standard deviation of each variable.

**FIGURE 8:**
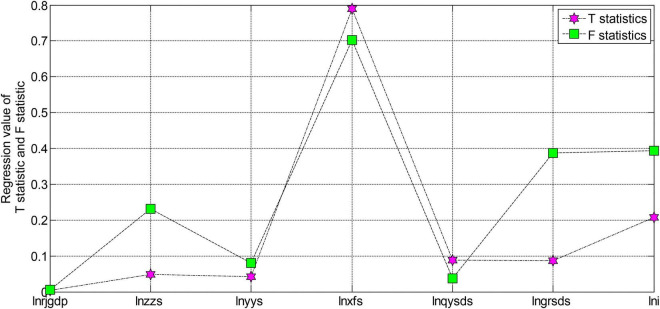
The regression results of the T statistic and F statistic of each variable of the model.

From the test results in Figures 5, 6, we can see that the ADF test value of each variable is greater than the critical value under the three significance levels. Therefore, the original variables are all non-stationary variables. The first-order difference form performs the unit root test again. After testing, the first-order difference form of the variables is all stationary variables. After the test, each variable is a first-order single integer variable. Next, we will perform a co-integration analysis on these first-order single-integral variables to determine the co-integration relationship. However, due to many variables and insufficient longitudinal extension of the sample, the sample is not long enough, so it is impossible to test these variables. However, using the sample data from 2003 to 2020 to regress the model has some guiding significance at some levels. The regression results of each variable of the model are shown in Figures 7, 8.

The residual of the model is a stationary series, indicating that there is indeed a certain cointegration relationship between these unstable variables. According to the T statistic value of each variable, it can be seen that in the coefficients of the entire estimation equation, the estimated coefficient of the variable of fixed asset investment in the whole society is not significant, and the estimated coefficients of business tax and corporate income tax are both significant at the significance level of 0.1. The estimated coefficients of value-added tax and employee personal income tax of tobacco companies are significant at the significance level of 0.05. The coefficients of the constant term, consumption tax, and time-variable are all significant at the significance level of 0.02. These insignificant and non-significant variables may be caused by too many variables in the model design and insufficient sample data length. However, in general, the regression results of the model are still more significant. The T statistic value is very high, and the explanatory degree of the variables involved in the model is also very high, reaching more than 95%. Therefore, the overall fit of this regression equation is still very good.

### Discussion

For enterprises with relatively weak competitiveness, taxation brings more pressure to them, which will affect the development of small and medium-sized enterprises; at the same time, the proportion of direct taxes mainly based on personal income tax and property tax is too small, which is not conducive to regulating our country. Over the last few years, the study shows that fewer taxes have increased in China, substantially affecting its economic growth (Zhang et al., 2019). China’s tobacco industry is a fast-growing industry, which largely depends on generating revenue to achieve financial stability (Kohrman et al., 2020). However, in the short term, at the current stage of my country’s economic and social development, it is a very difficult task to increase the proportion of the direct tax and reduce the proportion of turnover tax in a short period. A long-term task of tax reform is to rationally adjust the ratio of indirect tax and direct tax, break the tax structure dominated by turnover tax, and constantly adjust the ratio of the two to gradually form a dual-oriented tax system dominated by turnover tax and income tax.

And how to adjust the proportion of direct tax and indirect tax, on the one hand, we can increase the tax types of the income tax system by levying some new taxes such as social security tax, inheritance tax, gift tax, etc.; on the other hand, we must look at the solution to the problem from an overall perspective, that is, to achieve through increments, to convert a part of my country’s non-tax government revenue into direct tax in a certain way. Those who are divided equally can better contribute to economic growth.

Undoubtedly, tobacco prices and consumers’ earnings are crucial in driving tobacco consumption. The high affordability of tobacco products makes the consumers buy more products, thus increasing its market demand (Nargis et al., 2021). In particular, the aggressive adoption of an effective tax policy controls tobacco consumption, bringing numerous health benefits for consumers and the environment (Fuchs et al., 2019). However, the advantage of managing the taxes can only be understood when the consumer positively responds to the government policy. The effective fiscal policy (i.e., taxation) lowers tobacco consumption and escalates the government revenue, thereby influencing the nation’s growth (Hiscock et al., 2018). Perhaps, we need to make reasonable adjustments to the consumption tax rate. The current consumption tax adopts two forms: fixed and proportional taxes. The tax payable has a great relationship with the consumption tax rate and tax base formulated. In terms of tax rate adjustment, it is also possible to refer to the method of adjusting the scope of taxation to achieve both increase and decrease. For example, the tax rate of cosmetics can be adjusted reasonably, and the tax rate of ordinary car products can be reduced. The tax rate of products that run counter to environmental protection, such as lunch boxes and solid wood floors, should be increased for those products with non-renewable resources, scarce resources, and high environmental pollution. In this concern, to acquire healthy China, tobacco taxes need to be controlled and managed to gain the country’s economic and environmental sustainability (Goodchild and Zheng, 2019). Therefore, the consumption tax reform should be adjusted reasonably from the perspective of environmental protection and lack of resources.

## Conclusion

This paper constructs a composite index of individual income tax for tobacco company employees through the reasonable division of individual tax income. It uses the LMDI decomposition method to decompose the incremental decomposition and development speed decomposition of individual tax income from the national and regional levels. Whether it is the decomposition of the eastern, central, western, and northeastern regions, or the decomposition results from the country’s overall level, they all indicate that the aging effect is the main reason for hindering tax revenue. The decomposition results are specifically reflected in the fact that the aging of each region or country is increasing, the amount of change in tax revenue caused by aging is negative, the development speed indicator is less than 1, and a negative growth rate has occurred. The changes in tax structure and macro tax burden during the sample observation period do not have a definite trend but fluctuate up and down. The fluctuation of these two effects will cause changes in the same direction as the individual tax revenue. The specific manifestations are tax structure and macro tax.

Negative effects have a two-way effect on changes in personal tax revenue. The growth of tax structure and the macro tax burden will drive the growth of personal tax revenue, and the reduction of tax structure and the macro tax burden will hinder the growth of personal tax revenue. Through empirical analysis of the impact of different taxes on economic growth, we find that value-added tax, consumption tax, and personal income tax of tobacco company employees have a negative impact on China’s per capita GDP. It has a restraining effect on economic growth, with business tax and corporate income tax impacting China’s per capita GDP. Indeed, it is positive, which is conducive to China’s economic growth. The effect of value-added tax on economic growth is inseparable from the narrow scope of China’s value-added tax collection. The collection of corporate income tax can promote economic growth. Certain aspects are also affected by China’s actual economic conditions. The existence of tax collection methods, tax rates, and tax evasion does not fully reflect the original intention of narrowing the income gap. Therefore, it has a negative impact on China’s economic growth.

### Practical Contribution and Further Research

This paper mainly studies the impact of taxation on economic growth from the two aspects of macro tax burden and tax structure. It adopts medium-caliber macro tax burden data in the aspect of the macro tax burden. At the same time, the price level of GDP, the added value of per capita GDP, and time are used as control variables for analysis. In terms of tax structure, value-added tax, business tax, consumption tax, corporate income tax, and personal income tax are used as explanatory variables, and the total investment in fixed assets and time series of the whole society are used as control variables to analyze and study the impact of different taxes on economic growth. However, as far as the impact of taxation on economic growth is concerned, further research can be done from the regional composition of taxation amount to find out the different effects of taxation on economic growth in different regions, and then formulate different policies according to the impact of different regions. Therefore, this will also be the direction of further research in this paper in the future.

## Data Availability Statement

Publicly available datasets were analyzed in this study. The data is publicly available on China National Knowledge Infrastructure (CNKI). https://data.cnki.net/.

## Author Contributions

The author confirms being the sole contributor of this work and has approved it for publication.

## Conflict of Interest

The author declares that the research was conducted in the absence of any commercial or financial relationships that could be construed as a potential conflict of interest.

## Publisher’s Note

All claims expressed in this article are solely those of the authors and do not necessarily represent those of their affiliated organizations, or those of the publisher, the editors and the reviewers. Any product that may be evaluated in this article, or claim that may be made by its manufacturer, is not guaranteed or endorsed by the publisher.
